# Supplementary figures and images for: Regulation of microtubule dynamic instability by the carboxy-terminal tail of β-tubulin
Source: Life Sci Alliance. 2018 Apr 19;1(2):e201800054. doi: 10.26508/lsa.201800054 (PMC6022761; doi:10.26508/lsa.201800054)

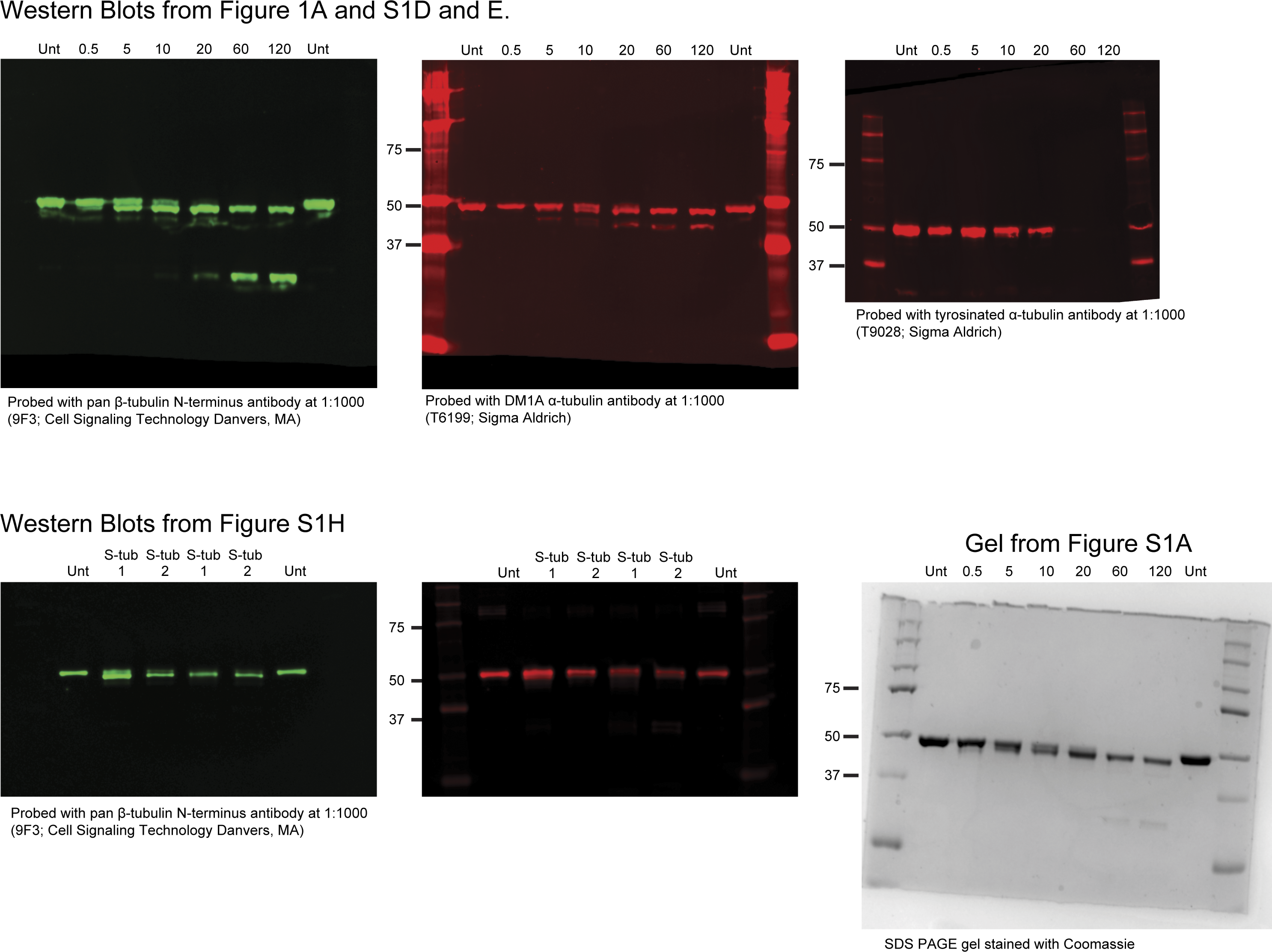

Supplement: Supplementary file 2 [file LSA-2018-00054_SdataF1.tif]
